# Relationship between Blood Pressure and Outcomes in Acute Ischemic Stroke Patients Administered Lytic Medication in the TIMS-China Study

**DOI:** 10.1371/journal.pone.0144260

**Published:** 2016-02-01

**Authors:** Wei Wu, Xiaochuan Huo, Xingquan Zhao, Xiaoling Liao, Chunjuan Wang, Yuesong Pan, Yilong Wang, Yongjun Wang, TIMS-CHINA investigators

**Affiliations:** 1 Department of Neurology, Beijing Tiantan Hospital, Capital Medical University, Beijing, China; 2 Department of neurology, Qilu Hospital of Shandong University, Jinan China & Braian Science Research Institute, Shandong University, Jinan, China; Stanford University, UNITED STATES

## Abstract

**Objective:**

Increased blood pressure (BP) management following acute ischemic stroke (AIS) remains controversial. This study aimed to identify the association between BP and clinical outcomes in AIS patients administered lytic medication in the TIMS-China (thrombolysis implementation and monitor of acute ischemic stroke in China) database.

**Methods:**

The sample comprised 1128 patients hospitalized within 4.5 hours (h) of AIS for intravenous recombinant tissue plasminogen activator (i.v. rt-PA) thrombolysis. Systolic BP (SBP) and diastolic BP (DBP) at baseline, 2 h and 24 h after treatment, and changes from baseline were analyzed. The study outcomes comprised a favorable outcome (modified Rankin Scale 0–1 at 90 days) and symptomatic intracerebral hemorrhage (SICH), analyzed using logistic regression, with low BP as the reference group.

**Results:**

Lower BP (baseline, 2 h, and 24 h) was beneficial in AIS patients and significantly related to a favorable outcome (P<0.05). A substantial BP decrease at 24 h after rt-PA thrombolysis was significantly associated with a favorable outcome compared with a moderate BP decrease (P = 0.0298). A SBP >160 mmHg 2 h after rt-PA thrombolysis was significantly associated with SICH compared with a SBP <140 mmHg (P = 0.0238). An increase or no change (>25 mmHg) in SBP was significantly associated with SICH (P = 0.002) compared with a small SBP decrease (1–9 mmHg).

**Conclusions:**

This study provides novel evidence that lower BP within the first 24 h is associated with a more favorable outcome and less frequent SICH in AIS patients administered lytic medication. Routine BP-lowering treatment should be considered in AIS patients following lytic medication.

## Introduction

The prevalence of hypertension ranges from 5 to 47% in men and 7 to 38% in women in the Asia Pacific region, and up to 66% of cardiovascular (CV) disease subtypes are attributed to hypertension[1]. Despite a rapid increase in the prevalence of hypertension in China, only 30% of individuals with hypertension are aware of their condition. Thus, proper blood pressure (BP)-lowering strategies will have an immense impact in this region, especially Asia.

Increased BP was observed in over 60% of the patients presenting with acute ischemic stroke (AIS)[2]. Favorable outcomes in AIS patients focus on decreases in stroke-induced disabilities. Thus, BP management is strongly recommended. Guidelines recommend intravenous (i.v.) BP medication to simplify treatment, as well as for convenience and effectiveness[3, 4] (PMID: 23370205). However, the optimal approach for BP-lowering management in AIS remains conflicting[3–5]. Furthermore, a high BP is beneficial to maintain blood flow in the ischemic brain; however, it may also be detrimental with regard to brain edema and hemorrhagic transformation (HT)[6].

The thrombolysis implementation and monitor of AIS in China (TIMS-China) registry was created to investigate the safety and efficacy of i.v. recombinant tissue plasminogen activator (rtPA) treatment within an extended time window (4.5 h) in Chinese AIS patients[7]. The patient data were obtained from the TIMS-China registry, and the objective of the post-hoc analysis focused on the correlation between BP management and outcomes in AIS patients.

## Materials and Methods

### Ethics Statement

The study protocol was approved by the Institutional Review Board at the Beijing Tiantan Hospital. All patients gave written informed consent to participate and the privacy of patients was strictly protected.

### Patient population

During the period of January 5, 2007 to July 31, 2010, all cases at 67 investigative sites in the Republic of China were prospectively registered in the TIMS-China Registry database. The patients were female or male, between 18 and 80 years of age, clinically diagnosed of ischemic stroke, and had underwent a computed tomography (CT) or magnetic resonance imaging (MRI) scan that ruled out hemorrhage, major ischemic infarction, or other non-ischemic diseases. Stroke symptoms presented for at least 30 min and did not significantly improve prior to treatment in the current study. The patients had no contraindication for thrombolysis therapy and provided informed consent for the treatment. The study was conducted in compliance with the protocol, the principles developed in the Declaration of Helsinki, the International Conference on Harmonisation Harmonised Tripartite Guidelines for Good Clinical Practice, and applicable regulatory requirements. The study protocol was approved by the Ethics Committee of Beijing Tiantan Hospital. The registry was independently monitored on a regular basis via the quality monitoring committee of the TIMS-China study. All study participants provided written informed consent prior to entering the study.

### Study design

The TIMS-China study comprised a prospective, multicenter, open-label, observational study conducted from January 5, 2007 to July 31, 2010 at 67 investigative sites in the Republic of China. The participants included in the trial were divided into 3-h and 3–4.5-h time-window groups according to the onset-to-needle time window. I.V. rtPA within 3 h is the only approved therapy for AIS. rtPA was licensed for AIS in China in 2004 based on the National Institute of Neurological Disorders and Stroke (NINDS) trial criteria. Consecutive AIS patients within the 4.5-h time window were screened at all participating centers following arrival. I.V. rtPA thrombolysis was performed in patients within 2 h of onset-to-door time window and was guided by plain CT scan according to the NINDS criteria.

### Efficacy assessments

The seated systolic blood pressure (SBP) and diastolic blood pressure (DBP) were measured at baseline, as well as 2 h and 24 h after treatment. A standard, validated, and calibrated traditional manual cuff sphygmomanometer in good condition was used. A random zero sphygmomanometer with blinded measurements or an automated device were not permitted. BP measurements were performed on the same arm. The accuracy of the BP measurements was increased by using the mean of three consecutive measurements approximately 2 min apart, as well as requiring the same individual to measure the BP at each visit. Vital signs, including the SBP, DBP, and pulse pressure, were considered the Safety Endpoints.

### Safety endpoint assessments

The SBP and DBP at baseline, 2 h and 24 h after treatment, and the corresponding changes from baseline were summarized with descriptive statistics for the entire population, as well as specific BP groups. The BP data for each patient at each time point assessed are available upon request.

For the correlation between BP (baseline, 2 h, and 24 h) and ICH based on imaging diagnosis at 24 h and 7 days after treatment or symptomatic intracerebral hemorrhage (SICH) within 36 h after treatment, a logistic regression analysis was performed to investigate the relationship between the binary variable (ICH based on imaging diagnosis/SICH, Yes or No) and each BP variable (baseline, 2 h, and 24 h).

### Characterization of SBP groups

The SBP change was classified into the following 4 groups of approximately equal size. Group 1 (n = 498 for a 2-h change and n = 401 for a 24-h change) comprised patients with no change or an increase in SBP. The patients with a decrease in SBP were divided into tertiles: Group 2 (n = 216 for a 2-h change and n = 240 for a 24-h change) comprised patients with a small decrease in SBP (1–9 mmHg for a 2-h change and 1–11 mmHg for a 24-h change); Group 3 (n = 195 for a 2-h change and n = 228 for a 24-h change) comprised patients with a moderate decrease in SBP (10–18 mmHg for a 2-h change and 12–24 mmHg for a 24-h change); and Group 4 (n = 194 for a 2-h change and n = 214 for a 24-h change) comprised patients with a substantial decrease in SBP (>19 mmHg for a 2-h change and >25 mmHg for a 24-h change).

### Statistical analyses

The outcomes of interest in the present study included a favorable outcome (mRS 0–1 at 90 days) and SICH (defined as a CT-documented hemorrhage that was temporally related to deterioration in the patient’s clinical condition in the judgment of the clinical investigator. SICH attributable to study medication was defined as a symptomatic hemorrhage that occurred within 36 h of treatment onset).

The mRS scores at 90 days after treatment are summarized using frequencies and percentages by treatment groups. For the correlation analysis between BP (SBP and DBP at baseline, 2 h, and 24h after rtPA; 2-h and 24-h BP change from baseline) and favorable outcome (mRS 0–1) at 90 days after treatment, Pearson’s correlation coefficients and the associated p values were determined. The Pearson’s correlation analysis was performed using the SAS procedure correction. The same statistical analyses were used for the correlation analysis between BP and good neurological recovery versus neurological deterioration during hospitalization. The baseline differences among the four SBP groups were compared using the χ2 method for categorical variables and one-way analysis of variance (ANOVA) with probability values for the linear trend for continuous variables.

## Results

A total of 1128 patients who received i.v. rtPA treatment were registered in the TIMS-China database. The baseline characteristics and clinical outcomes are shown in Table 1. The patients’ mean age was 63.48 years, and 60.99% of the patients were men. Most patients (>59.93%) were non-smokers. Regarding medical conditions, 59.13% of the patients had a history of hypertension, 9.04% had a history of TIA, and 17.38% had diabetes. During the treatment period, 140 patients (12.41) exhibited an ICH based on imaging diagnosis at 24 h after treatment. Sixty-two patients (5.50%) died within 7 days after treatment.

**Table 1:** Baseline characteristics and clinical outcome.

Category	Value (%)
Gender	
Male (%)	688 (60.99)
Female (%)	440 (39.01)
Age	
Mean±SD	63.48±11.34
Median (Q1-Q3)	64.00 (56.00–73.00)
Min;Max	22.00;89.00
Atrial fibrillation	202 (17.91)
TIA history	102 (9.04)
Hypertension	667 (59.13)
Diabetes	196 (17.38)
Hyperlipidemia	73 (6.47)
Current smoker (last 6 months)	387 (34.31)
Previous smoker (before 6 months)	452 (40.07)
Stroke history	208 (18.44)
Glucose (mmol/L)	n = 1093
Mean±SD	7.72±3.03
Median (Q1-Q3)	6.90 (5.90–8.50)
Min;Max	3.50;33.52
INR (n = 1102)	
Mean±SD	1.02±0.16
Median (Q1-Q3)	1.00 (0.93–1.08)
Min;Max	0.68;4.20
Oral/i.v. antihypertensive agents 24 h pre-thrombolysis	432 (38.30)
Oral/i.v. antihypertensive agents in 7 days post-thrombolysis	370 (32.80)
Stroke onset to initiation of thrombolysis (h)	
Mean±SD	2.82±0.80
Median (Q1-Q3)	2.83 (2.33–3.28)
Min;Max	0.00;4.50
Baseline NIHSS	
Mean±SD	12.19±6.86
Median (Q1-Q3)	11.00 (7.00–16.00)
Min;Max	0.00;40.00
Dose of rt-PA(mg/kg)	
Mean±SD	0.86±0.10
Median (Q1-Q3)	0.90 (0.86–0.90)
Min;Max	0.14;1.33
SICH defined by NINDS	61 (5.41)
Hemorrhagic transformation (HT) in 24 h after thrombolysis	140 (12.41)
Deaths by day 7	62 (5.50)
Deaths by day 90	115 (10.39)
90 day mRS in (0,1)	529 (47.87)
Pre-thrombolysis SBP	
Mean±SD	148.03±20.95
Median (Q1-Q3)	150.00 (133.00–162.00)
Min;Max	88.00;230.00
SBP 2 h (±15 min) post-thrombolysis	
Mean±SD	143.59±20.33
Median (Q1-Q3)	142.00 (130.00–159.00)
Min;Max	56.00;247.00
SBP 24 h (±2 h) post-thrombolysis	n = 1106
Mean±SD	138.98±19.88
Median (Q1-Q3)	140.00 (126.00–152.00)
Min;Max	80.00;213.00

Abbreviations—h: hours; i.v.: intravenous; min: minimum; max: maximum; NIHSS: National Institutes of Health Stroke Scale; NINDS: National Institute of Neurological Disorders and Stroke; rt-PA: recombinant tissue plasminogen activator; SD: standard deviation; TIA: transient ischemic attack

### Associations among SBP and favorable outcomes

The associations between the 0- to 24-h profiles of the mean SBP and favorable outcomes at day 90 (mRS 0–1) were examined. After adjustment for the known baseline predictors (sex, age, acetylsalicylic acid medication history, onset to treatment interval, National Institutes of Health Stroke Scale, and ASPECT), SBP was inversely associated with a favorable outcome at day 90 in the rtPA-treated patients (Table 2). Overall, the sample means of SBP were numerically lower in the patients who had favorable outcomes (mRS 0–1).

**Table 2:** BP and favorable outcome (mRS 0–1 at 90 days).

	Total	
90 day (mRS 0–1)	OR (95%CI)	p
**Baseline SBP**	0.98 (0.98–0.10)	<0.0001
**2-h SBP**	0.98 (0.97–0.99)	<0.0001
**24-h SBP**	0.98 (0.97–0.98)	<0.0001
**0- to 2-h SBP change**	0.10 (0.99–1.01)	0.4836
**0- to 24-h SBP change**	0.10 (0.99–1.00)	0.1688

Abbreviations—BP: blood pressure; CI: confidence interval;

mRS: modified rankin scale; OR: odds ratio; SBP: systolic blood pressure

The associations between the different SBP groups and favorable outcomes were further examined (Table 3). Compared with the high baseline SBP group (>160 mmHg), a normal baseline SBP (140–160 mmHg; P = 0.0105, OR = 1.532, 95%CI 1.11–2.13) and a low baseline SBP (<140 mmHg; P<0.0001, OR = 2.23, 95%CI 1.58–3.15) were significantly associated with a favorable outcome. For the baseline SBP, a lower baseline SBP was significantly associated with a favorable outcome(P<0.0001, OR = 0.98, 95%CI 0.98–0.99).

**Table 3:** BP grouping of different time points & favorable outcome (mRS 0–1 at 90 days).

BP time points and grouping	Number of Events/At Risk (%)	Crude		Multivariate-Adjusted^Δ^	
		OR (95%CI)	p	OR (95%CI)	p
**A_SBP**	529/1105 (47.87)				
**<140**	196/346 (56.65)	2.12(1.58–2.84)	<0.0001	2.23 (1.58–3.15)	0.0000
**140–160**	183/366 (50.00)	1.62 (1.21–2.16)	0.0011	1.53 (1.11–2.13)	0.0105
**>160**	150/393 (38.17)	1 (Reference)	…	1 (Reference)	…
**TPA2H_SBP**	529/1103 (47.96)				
**<140**	255/440 (57.95)	3.048 (2.22–4.19)	<0.0001	3.12 (2.16–4.51)	0.0000
**140–160**	189/390 (48.46)	2.08 (1.50–2.88)	<0.0001	2.05(1.42–2.95)	0.0001
**>160**	85/273 (31.14)	1 (Reference)	…	1 (Reference)	…
**TPA24H_SBP**	528/1083 (48.75)				
**<140**	304/530 (57.36)	2.69 (1.89–3.83)	<0.0001	2.91 (1.94–4.37)	0.0000
**140–160**	163/370 (44.05)	1.58 (1.09–2.28)	0.0160	1.74 (1.15–2.63)	0.0088
**>160**	61/183 (33.33)	1 (Reference)	…	1 (Reference)	…
**SBP change 0–2 h**	529/1103 (47.96)				
**Substantial decrease (>19 mmHg)**	99/194 (51.03)	1.27 (0.85–1.89)	0.2443	1.38 (0.88–2.16)	0.1616
**Moderate decrease (10–18 mmHg)**	88/195 (45.13)	1 (Reference)	…	1 (Reference)	…
**Small decrease (1–9 mmHg)**	106/216 (49.07)	1.17 (0.80–1.73)	0.4237	1.14 (0.74–1.77)	0.5470
**Increase/no change**	236/498 (47.39)	1.10 (0.79–1.53)	0.5917	1.21 (0.84–1.76)	0.3117
**SBP change 0–24 h**	528/1083 (48.75)				
**Substantial decrease (>25 mmHg)**	118/214 (55.14)	1.66 (1.14–2.42)	0.0083	1.60(1.05–2.44)	0.0298
**Moderate decrease (12–24 mmHg)**	97/228 (42.54)	1 (Reference)	…	1 (Reference)	…
**Small decrease (1–11 mmHg)**	120/240 (50.00)	1.35 (0.94–1.95)	0.1063	1.29 (0.86–1.94)	0.2194
**Increase/no change**	193/401 (48.13)	1.25 (0.90–1.74)	0.1770	1.23 (0.85–1.78)	0.2712

Abbreviations—BP: blood pressure; CI: confidence interval; mRS: modified rankin scale; OR: odds ratio; SBP: systolic blood pressure; TPA: Tissue Plasminogen Activator

^**Δ**^Adjusted for sex, age, acetylsalicylic acid medication history, onset to treatment interval, National Institutes of Health Stroke Scale (NIHSS), and the Alberta Stroke Program Early CT Score (ASPECTS)

For the 2-h SBP, a lower SBP was significantly associated with a favorable outcome. A normal 2-h SBP (140–160 mmHg; P = 0.0001, OR = 2.048, 95%CI 1.42–2.95) and a low 2-h SBP (<140 mmHg; P = 0.0000, OR = 3.120, 95%CI 2.160–4.51) were significantly associated with a favorable outcome compared with the high 2-h SBP group (>160 mmHg).

For the 24-h SBP, a lower SBP was also significantly associated with a favorable outcome. Compared with the high 24-h SBP group (>160 mmHg), a normal 24-h SBP (140–160 mmHg; P = 0.0105, OR = 1.74, 95%CI 1.15–2.63) and a low 24-h SBP (<140 mmHg; P = 0.0000, OR = 2.91, 95%CI 1.94–4.37) were significantly associated with favorable outcomes. Furthermore, between 0 to 24 h, a substantial decrease (>25 mmHg) in SBP was significantly associated with a favorable outcome (P = 0.0298, OR = 1.60, 95%CI 1.05–2.44) compared with a moderate decrease (12–24 mmHg).

### Association between SBP and SICH

The associations between the 0- to 24-h profiles of the mean SBPs and SICH were examined (Table 4). For the baseline SBP, a normal baseline SBP (140–160 mmHg) was significantly associated with SICH compared with the high baseline SBP group (> 160 mmHg) (P = 0.0347, OR = 2.03, 95%CI 1.05–3.90). For the 2-h SBP, a high 2-h SBP (>160 mmHg) was significantly associated with SICH compared with the low 2-h SBP group (<140 mmHg; P = 0.0238, OR = 2.24, 95%CI 1.11–4.50). Furthermore, there was a tendency towards more frequent cases of SICH with a higher SBP at 2 h in the rtPA-treated patients(3.56% for low 2-h SBP, 5.30% for normal and 8.57% for high).

**Table 4:** BP grouping of different time points and SICH.

BP time points and grouping	Number of Events/At Risk (%)	Crude		Multivariate-Adjusted^Δ^	
		OR (95%CI)	p	OR (95%CI)	p
**A_SBP**	61/1128 (5.41)				
**<140**	17/353 (4.82)	1 (Reference)	…	1 (Reference)	…
**140–160**	28/375 (7.47)	1.60 (0.86–2.97)	0.1407	1.59 (0.82–3.07)	0.1699
**>160**	16/400 (4.00)	0.82 (0.41–1.66)	0.5857	0.78 (0.37–1.65)	0.5224
**TPA2H_SBP**	61/1126 (5.42)				
**<140**	16/450 (3.56)	1 (Reference)	…	1 (Reference)	…
**140–160**	21/396 (5.30)	1.52 (0.78–2.95)	0.2178	1.33 (0.67–2.65)	0.4232
**>160**	24/280 (8.57)	2.54 (1.33–4.88)	0.0050	2.24 (1.11–4.50)	0.0238
**TPA24H_SBP**	52/1106 (4.70)				
**<140**	25/542 (4.61)	1 (Reference)	…	1 (Reference)	…
**140–160**	15/377 (3.98)	0.86 (0.45–1.65)	0.6435	0.70 (0.35–1.42)	0.3228
**>160**	12/187 (187)	1.42 (0.70–2.89)	0.3345	1.34 (0.62–2.90)	0.4500
**SBP change 0–2 h**	61/1126 (5.42)				
**Substantial decrease (>19 mmHg)**	6/199 (3.02)	1 (Reference)	…	1 (Reference)	…
**Moderate decrease (10–18 mmHg)**	11/197 (5.58)	1.90 (0.69–5.25)	0.2143	1.74 (0.62–4.85)	0.2935
**Small decrease (1–9 mmHg)**	5/220 (2.27)	0.75 (0.23–2.50)	0.6368	0.76 (0.23–2.58)	0.6642#
**Increase/no change**	39/510 (7.65)	2.66 (1.11–6.39)	0.0283	2.31 (0.95–5.63)	0.0650*
**SBP change 0–24 h**	52/1106 (4.70)				
**Substantial decrease (>25 mmHg)**	8/220 (3.64)	1 (Reference)	…	1 (Reference)	…
**Moderate decrease (12–24 mmHg)**	9/233 (3.86)	1.06 (0.40–2.81)	0.8996	0.95 (0.35–2.54)	0.9119
**Small decrease (1–11 mmHg)**	13/242 (5.37)	1.50 (0.61–3.70)	0.3744	1.381 (0.55–3.49)	0.4949
**Increase/no change**	22/411 (5.35)	1.50 (0.66–3.43)	0.3376	1.285 (0.55–3.00)	0.5620

Abbreviations—BP: blood pressure; CI: confidence interval; OR: odds ratio; SBP: systolic blood pressure; SICH: symptomatic intracerebral hemorrhage; TPA: Tissue Plasminogen Activator

^**Δ**^Adjusted for sex, age, acetylsalicylic acid medication history, onset to treatment interval, National Institutes of Health Stroke Scale (NIHSS), and ASPECT

For SBP changes between 0 to 2 h, an increase or no change (>25 mmHg) in SBP was significantly associated with SICH (P = 0.002) compared with a small decrease in SBP (1–9 mmHg). Furthermore, a tendency towards more frequent cases of SICH was associated with an increase or no change (>25 mmHg) in SBP compared with a substantial decrease in SBP after rtPA (P = 0.0650, OR = 2.31, 95%CI 0.95–5.63).

## Discussion

More than 7 million stroke cases occur per year in China, and approximately 65% of these cases comprise ischemic stroke[8]. However, less than 10% of patients with AIS receive thrombolytic therapy[9]. This rate is lower than the rate of thrombolysis in Europe[10]. To the best of our knowledge, the current study represents the first investigation of the safety and outcomes of thrombolysis in Chinese patients with AIS.

High BP occurs in approximately 80% of patients with AIS[11] and typically decreases over the subsequent 7 days[12]. Several observational studies have examined the relationship between high initial BP and clinical outcome. A higher post stroke BP has been associated with unfavorable clinical outcomes in several studies[13, 14] and favorable outcomes in other studies[15, 16]. Furthermore, other studies have suggested the presence of a J- or U-curve phenomenon between post stroke BP and functional outcome[11, 17, 18]. The ‘Efficacy of Nitric Oxide in Stroke’ trial tested whether transdermal glyceryl trinitrate, a nitric oxide donor that lowers blood pressure, is safe and effective in improving outcome after acute stroke. The results of this trial showed that in patients with acute stroke and high blood pressure, transdermal glyceryl trinitrate lowered blood pressure and had acceptable safety but did not improve functional outcome. The ENOS trail showed no evidence to support continuing prestroke antihypertensive drugs in patients in the first few days after acute stroke[19].

These previous studies have failed to address post stroke BP and functional outcome following lytic medication in 4.5 h, which has resulted in a critical gap in our understanding of the role of BP in AIS. To the best of our knowledge, the current study provides the first evidence that BP-lowering treatments is beneficial for AIS patients following lytic medication.

In this study, BP control was necessary for the AIS patients, especially the patients who received i.v. anti-hypertensive medication. Our findings are consistent with previous studies conducted with AIS patients administered rt-PA treatment[20]. The baseline SBP and the range of decreases in BP were related to favorable outcomes in the current population of AIS patients. These findings are similar to a trial SITS thrombolysis register study, which indicated there was a strong association between high SBP after thrombolysis and poor outcome[21].

Our findings also demonstrated that a higher 2-h SBP was related to SICH. These results were consistent with the second European Cooperative Acute Stroke Study (ECASS-II) trial, which indicated that hemorrhagic transformation within the first 7 days and favorable outcome were independently associated with BP dynamics within the first 24 h after an AIS in patients treated with thrombolysis[20]. However, the current findings provide novel evidence that a normal BP was related to an increased risk of SICH compared with a high BP at baseline. Thus, we further examined the relationship between BP changes between 0 and 2 h and SICH and determined that an increase or no change in BP during this time period was significantly related to an increased prevalence of SICH compared with a small decrease in BP. An increase or no change in BP between 0 to 2 h was also associated with a tendency towards an increased risk of SICH compared with a substantial decrease in SBP. These findings suggest that proper control of SBP for the first 2 h is of critical importance to decrease the risk of SICH.

Our data provided the evidence that lower BP is beneficial for AIS patients following lytic medication in 4.5 h. These results added the clinical experience of BP control for Chinese patients with AIS.

Finally, there are several limitations that should be considered in the interpretation of these results. This study did not comprise a randomized, placebo-controlled trial. Nevertheless, the current study provides novel evidence from a large sample of patients that indicates the critical role of SBP during the initial hours following AIS.

## Conclusions

A lower BP within the first 24 h following AIS is associated with a more favorable outcome and less SICH following lytic medication. Routine BP-lowering treatments should likely be considered for AIS patients following lytic medication.
